# Applicability of major histocompatibility complex DRB1 alleles as markers to detect vertebrate hybridization: a case study from Iberian ibex × domestic goat in southern Spain

**DOI:** 10.1186/1751-0147-54-56

**Published:** 2012-09-24

**Authors:** Samer Alasaad, Joerns Fickel, Luca Rossi, Mathieu Sarasa, Buenaventura BenÃ-tez-Camacho, José E Granados, Ramón C Soriguer

**Affiliations:** 1 Estación Biológica de Doñana, Consejo Superior de Investigaciones Científicas (CSIC), Avda. Américo Vespucio s/n, 41092, Sevilla, Spain; 2 Institute of Evolutionary Biology and Environmental Studies (IEU), University of Zürich, Winterthurerstrasse 190, 8057, Zürich, Switzerland; 3 Research Group Evolutionary Genetics, Leibniz-Institute for Zoo and Wildlife Research, Alfred-Kowalke-Str. 17, D-10315, Berlin, Germany; 4 Dipartimento di Produzioni Animali, Epidemiologia ed Ecologia, Università degli Studi di Torino, Via Leonardo da Vinci 44, I-10095, Grugliasco, Italy; 5 Grupo Biología de las especies cinegéticas y plagas (RNM-118), Avda. Américo Vespucio s/n, Sevilla, 41092, Spain; 6 c/Maestro Francisco Carmona, 9-2, 14810, Carcabuey, Córdoba, Spain; 7 Espacio Natural de Sierra Nevada, Carretera Antigua de Sierra Nevada, Km 7.5, Córdoba, Spain

**Keywords:** Hybridization, MHC alleles, Capra pyrenaica, Capra hircus, Wildlife conservation

## Abstract

**Background:**

Hybridization between closely related wild and domestic species is of great concern because it can alter the evolutionary integrity of the affected populations. The high allelic variability of Major Histocompatibility Complex (MHC) loci usually excludes them from being used in studies to detect hybridization events. However, if a) the parental species don’t share alleles, and b) one of the parental species possesses an exceptionally low number of alleles (to facilitate analysis), then even MHC loci have the potential to detect hybrids.

**Results:**

By genotyping the exon2 of the MHC class II DRB1 locus, we were able to detect hybridization between domestic goats (*Capra hircus*) and free-ranging Iberian ibex (*Capra pyrenaica hispanica*) by molecular means.

**Conclusions:**

This is the first documentation of a *Capra pyrenaica* × *Capra hircus* hybridization, which presented us the opportunity to test the applicability of MHC loci as new, simple, cost-effective, and time-saving approach to detect hybridization between wild species and their domesticated relatives, thus adding value to MHC genes role in animal conservation and management.

## Background

Numerous genetic studies have shown that hybridization do not only occur more commonly than originally thought but also across the entire animal kingdom [1-4]. In particular, hybridization occurs between wild species and their domestic counterparts [5,6], as well as between native and introduced species [4,7,8].

If the fitness of the hybrid-offspring is greater than that of the offspring from each parent species, hybridization on one hand may function as mode of speciation while on the other hand disrupt local adaptations, leading to population decline and loss of biodiversity [9,10]. The list of consequences observed after hybridization between domestic and wild species is extensive and may include reduced fitness of F1 and F2 generations, accelerated growth rate with subsequent skeletal malformations, increased agonistic behaviour, decreased predator avoidance behaviour, and unpredictable effects on animals’ resistance to parasites [11-13].

While in the past hybridizations were considered to be rare events, nowadays they are increasingly detected because molecular methods often show hybridization despite unchanged phenotype [14]. Because mammalian mitochondrial DNA (mtDNA) is maternally inherited, the detection of mtDNA haplotypes of one species in yet another, morphologically distinct species indicates introgression and thus past hybridization followed by numerous backcrosses [5,10,15,16]. Nevertheless, mtDNA can only detect hybridization along matrilines while paternal hybrid offspring remains undetected. Moreover, mtDNA haplotypes may not be monophyletic within species because of ancestral lineage sorting in addition to hybridization [17]. In the opposite case, Y-linked haplotype markers will only be useful to detect male hybrid offspring as they follow the paternal lineage [18] and thus, results inferred from mitochondrial DNA and Y-chromosome are sometimes discordant. Due to their biparental inheritance, microsatellites are thus better suited to detect hybridization events, and therefore have been established and used for many animal species [19,20]. Nevertheless, for the eventual detection of hybridization, the use of microsatellites requires additional efforts such as standardization of allele lengths for both species and for the cross-species amplification in the hybrids [21]. They also require the use of internal genotyping controls when performing PCR amplifications to overcome the possible lack of consistency in allele sizes across different analytical instruments and running conditions [22]. A different approach, besides the use of mtDNA and microsatellite loci, is the exploration of the species specificity of coding sequences. To our knowledge, the genes of the Major Histocompatibility Complex (MHC) have not yet been used to detect hybridization events between vertebrate species.

In vertebrates, the MHC is vital for foreign antigen recognition and the immune response to infections [23]. Some of its genes are among the most polymorphic loci of the vertebrate genomes [23] displaying high levels of allelic diversity [24]. So far, at least six models have been suggested to explain the maintenance of MHC variability, the two most prominent ones being a) balancing selection and b) the rare allele model [25]. Interestingly, domestic species often have higher than expected levels of MHC diversity, given their domestication history [26]. On the other hand, many endangered species exhibit a low degree of MHC polymorphism caused by severe population bottlenecks in their history [27]. Low MHC variability may also stem from the social organisation of a species [28], which may result in low transmission rates of infectious diseases [29] generating low selection pressure for high variability. Hence, hybridizations between species with high MHC allelic variability and species with low MHC variability may be detected by studying MHC allele distribution, given that there are no shared alleles.

There are several reports about hybridizations in caprine species. Hybrids between several wild caprine species have been reported and hybrids between domestic goat and wild caprine species were described as being fertile and having a reduced predator and human avoidance [30]. In few cases, hybrids between the Alpine ibex (*Capra ibex ibex*) and domestic goats (*Capra hircus*) were reported for both crosses (wild male × domestic female; wild female × domestic male) and as occurring both in captivity [31] and in the wild [32]. However, to our knowledge, neither a general report nor a molecular data based report has so far presented hybridization between Iberian ibex (*Capra pyrenaica*) and domestic goat in the wild.

In this paper we aim to (i) present the first cases of free-ranging Iberian ibex × domestic goat hybrids, and want to (ii) demonstrate the suitability of MHC loci (under certain conditions) as a molecular tool for the detection of hybridization between species with strongly differing allelic variability at these loci.

## Methods

### The Iberian ibex

The Iberian ibex is an endemic species of the Iberian Peninsula, which has recently re-colonized northern Portugal [33]. Besides its biological and ecological value, the Iberian ibex is also a much sought after big game trophy, and hence has a high socio-economic value [34]. Although *C. pyrenaica* is of “least concern” in the IUCN Red List, there are increasing awareness and ongoing conservation efforts in Spain, especially because of outbreaks of *Sarcoptes* mite in the populations of Southern Spain [35,36]. Partial sequencing of exon2 of the MHC class II DRB1 gene revealed that the Iberian ibex has a remarkably low level of genetic variation at this locus, with only six different alleles [37].

### History of hybrids

**Figure 1:**
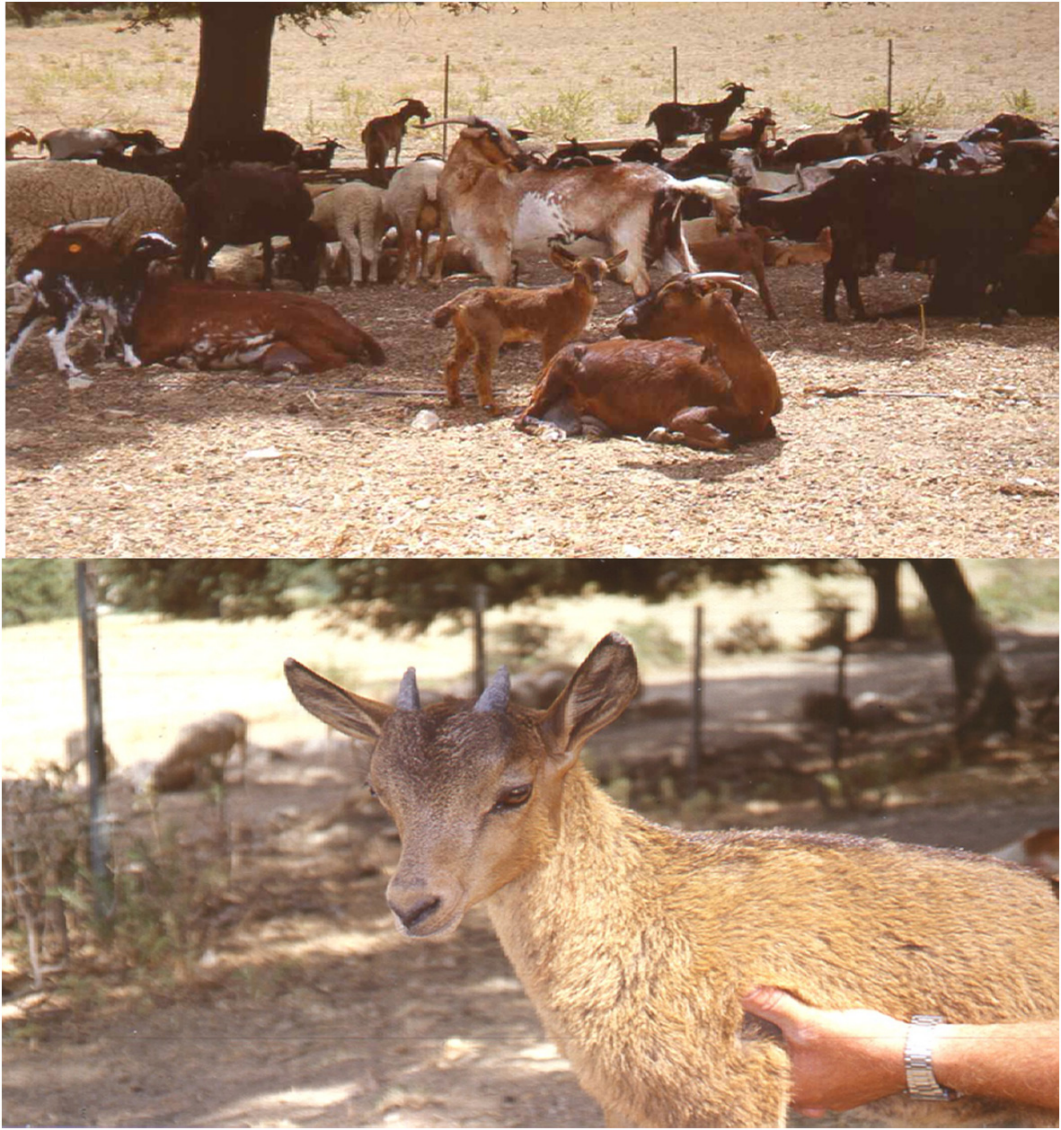
Picture of Iberian ibex x domestic goat hybrid calf with its domestic goat mother (up) and alone (down) in the Mountain of Rute-Priego (Cordoba, Spain).

In November 1996, a male Iberian ibex from the Rute-Priego Mountains (Cordoba, Spain) entered into a domestic goat herd (mainly Granadinas breed) on the Dehesa Bichira property. Under the acquiescence of the shepherd, who presumed that hybrid goatlings would be “super-goats” (heterosis enhanced phenotypes) the Iberian ibex mated with at least 12 domestic goat females, which gave birth to 15 hybrid offspring in March 1997 (Figure 1). However, the growth rate of the hybrids was similar to that of the domestic goatlings and the failed “super-goats” were slaughtered in October 1997. This situation presents a unique opportunity to validate a new tool for the detection of hybridization.

### Collection of hybrid samples and DNA extraction

Blood samples were collected, by authorized veterinarian during the annual Animal Health Control Program of Spanish Ministry of Agriculture, from two hybrids in May 1997 and kept, by chance, in 96% ethanol until summer 2011, when DNA was extracted. We also collected blood samples from 29 arbitrarily chosen domestic goats from the same area to investigate the genetic diversity at the exon2 of MHC class II DRBI and to confirm the absence of either one of the six *Capra pyrenaica* MHC DRB1 alleles from the domestic goat population. Due to the long preservation period of the two hybrids stored blood we used a DNA extraction protocol adapted to non-invasive faecal samples [38]. An aliquot (~200 μl) of both blood samples (mixed with ethanol) was shortly centrifuged, supernatants were removed and the precipitate was dried at 45°C for 30 min. Extractions were then carried out in a separate room exclusively dedicated to low copy number DNA samples. Two blanks (reagents only) were included to monitor for contamination.

### Ethics

Blood samples were collected during the annual Animal Health Control Program of Spanish Ministry of Agriculture. All animals were alive until, few month later, the owner slaughtered all the annual production for commercial purposes. Consent from owners was obtained for doing our research.

### PCR amplification and sequencing

Exon2 of MHC class II DRB1 was amplified by a two round semi-nested PCR (24), with some modification as follows: PCR I (pre-amplification): PCR reaction mixture (30 μl) consisted of 2 μl gDNA (25–100 ng/μl), 0.25 μM of each primer (DRB1.1 and GIo; [39]), 0.12 mM of each dNTP, 3 μl of 10x PCR buffer (Bioline), 1.5 mM MgCl_2_, 0.4% BSA, 1.5 μl DMSO, and 0.2 μl (5 U/μl) Taq polymerase (Bioline). Samples were subjected to the following thermal profile for amplification in a 2720 thermal cycler (Applied Biosystems): initial denaturing (4 min, 94°C), 15 cycles (60 s, 94°C; 60 s, 60°C; 50 s, 72°C), and final elongation (5 min, 72°C). PCR blanks (reagents only) were included.

PCR II (semi-nested): components, their concentrations, and thermal profile were similar to PCR I, except we used 2 μl of PCR I-product as template, substituted primer GIo by primer DRB1.2 [40], increased the annealing temperature to 65°C, and increased the number of cycles to 30. PCR blanks (reagents only) were likewise included.

PCR products were purified using the QIAquick PCR Purification Kit (Qiagen) and directly sequenced separately from both directions, applying the Big Dye® Terminator cycle sequencing kit v1.1 (Applied Biosystems, Darmstadt, Germany). Fragment separation and analysis were performed on an ABI model A3130*xl* Genetic Analyzer using the software Sequencing Analysis v.5.2 (ABI). Newly obtained DNA sequences were aligned, visually checked and edited using the software BioEdit v.7.0.9 [39].

## Results

PCR was successful in both 13-years-old blood samples, evidenced by a 237 bp long MHC class II DRB1 exon2 fragment, amplified by primer pair 2 (DRB1.1 and DRB1.2). Sequencing of the fragments revealed that both hybrids were heterozygous at this locus. One allele was identical to the Iberian ibex allele Capy-DRB1*5 ([37]; GenBank accession number AF461696). But because Amills *et al.*[37] had only genotyped 43 individuals, we carried out a complementary study on 160 wild Iberian ibex and verified the limited number of MHC class II DRB1 exon2 alleles in this species by direct sequencing, yielding again only the six already known alleles [41]. Based on the absence of allele Capy-DRB1*5 from domestic goats we identified this allele as the one inherited paternally from the Iberian ibex. The other allele then had to be the maternally inherited one from the domestic nanny goat. Its sequence was identical to the domestic goat MHC class II DRBI exon2 allele *Capra hircus*-DRB*13 ([42]; GenBank accession AB008358), which is not present in *Capra pyrenaica*.

## Discussions

Theoretically, hybridization would be often possible because space use of wild and domestic goats overlaps frequently. However, the only shortly overlapping interval between the mating seasons of wild and domestic goats, the mating aversion of wild females to domestic males and increased prenatal mortality rate are limiting factors for hybridization between wild and domestic populations [30]. Hybridizations have also been allowed or favoured as an attempt to improve livestock productivity or they resulted from translocations to zoological parks or reserves [15,30].

This is, to our knowledge, the first molecular documentation of a *Capra pyrenaica* × *Capra hircus* hybridization. Because of seasonal altitudinal migrations of wild goats and because of traditional pastoralism in mountainous environments, direct contact between ibexes and domestic goats can be expected to occur regularly. Such contacts might be more frequent during the summering of livestock in high parts of the mountain range and in lower parts during the rut in autumn-winter time, when animals look for mates and for food to bear adverse weather condition [30,43]. However, the close co-occurrence of domestic livestock and Iberian ibex is the main threat to *Capra pyrenaica* conservation [34,35], because domestic livestock might transmit diseases to Iberian ibex [44] and competes for resources [45]. Curiously, the risk of hybridization between Iberian ibex and domestic goat has so far been neglected. Nevertheless, hybridizations pose direct and indirect risks. Direct risks, because they may divert the evolutionary trajectory of affected populations and thus may even lead to extinction of formally allopatric taxa that are not reproductively isolated [46]. Indirect risk because they may compromise the resistance of animals to parasites [13]. For example, between 1901 and 1953 a herd of hybrids was bred using domestic goat (*Capra hircus*), Alpine ibex (*C. ibex*), west caucasian tur (*C. caucasica*) and Nubian ibex (*C. nubiana*) and released in the High Tatra mountains (30). But instead of having generated the best trophy game, this herd was quickly eradicated by an epidemic [30].

Numerous populations of Iberian ibex are also already challenged or threatened by contact-transmitted pathogens and by their susceptibility to epidemic outbreaks, of for example, *Sarcoptes scabiei*[47]. Thus, increased awareness and management consideration are required to control the risks of co-occurrence and thus of potential hybridizations between Iberian ibex and domestic goats.

Conserving the genetic integrity of the Caprinae as recommended by the IUCN Caprine Specialist Group requires appropriate reintroduction policies and adequate management programs to avoid hybridisation and, if occurring, to facilitate the removal of hybrids from sites of occurrence [32]. In order to do the latter, hybrids have to be identified unambiguously.

Cautions should be taken into account (i) in the presence of cross-species evolution in the studied species: commonly, cross-species evolution is characterized by the existence of phylogenetically very similar alleles in closely related species or if the species involved are in the host-spectrum of the same pathogens, and hence the occurrence of identical MHC alleles in other vertebrate hybridization systems cannot be excluded [48]; (ii) because recombination effects at MHC loci may also complicate the molecular identification of hybrids (particularly in ancient hybrids) [49].

Even though the blood samples had been stored in ethanol for 13 years, exon2 of MHC class II DRB1 was still amplifiable from them. The reasons behind that were likely (a) the shortness of the amplicon (237 bp) and (b) the use of a semi-nested PCR, which increases the amount of low copy number template because the products of the first amplification are used as templates for the subsequent PCR [50].

This is the first report of hybridization between Iberian ibex and domestic goat. Notwithstanding, the rangers of the Sierra Nevada Natural Space in Spain informed us that they already detected and removed ibex x domestic goat hybrids: one offspring and one adult male in 1997 (Additional files 1 and 2). This highlighted that hybridizations between both *Capra* species appear to occur frequently, and hence an appropriate molecular tool could be of vital interest for the conservation of the Iberian ibex.

## Conclusions

Our study represent the first documentation of a *Capra pyrenaica* × *Capra hircus* hybridization. Due to the fact that opportunity presented us a known hybrid between *Capra* species, we were able to test the applicability of MHC loci as new, simple, cost-effective, and time-saving approach to detect hybridization between wild species and their domesticated relatives, thus adding value to MHC genes role in animal conservation and management.

## Competing interests

The authors declare that they have no competing interests.

## Authors’ contributions

SA, CVB and RCS designed the study. JF, LR and MS executed the molecular analysis. SA, JF, LR, MS, CVB and RCS drafted the manuscript. All authors read and approved the final manuscript.

## Supplementary Material

Additional file 1 Figure S1. Picture of Iberian ibex x domestic goat hybrid calf in the Mountain of Sierra Nevada in 1997. Click here for file

Additional file 2 Figure S2. Picture of Iberian ibex x domestic goat hybrid adult in the Mountain of Sierra Nevada in 1997. Click here for file

## References

[B1] Mallet J Hybridization as an invasion of the genome Trends Ecol Evol 2005 20 229 237 10.1016/j.tree.2005.02.010 16701374

[B2] Arnold ML Meyer A Natural hybridization in primates: one evolutionary mechanism Zoology 2006 109 261 276 10.1016/j.zool.2006.03.006 16945512

[B3] Salzburger W Baric S Sturmbauer C Speciation via introgressive hybridization in East African cichlids? Mol Ecol 2002 11 619 625 10.1046/j.0962-1083.2001.01438.x 11918795

[B4] Cortés-Ortiz L Duda TF Canales-Espinosa D Garcia-Orduna F Rodriguez-Luna E Bermingham E Hybridization in large-bodied new world primates Genetics 2007 176 2421 2425 10.1534/genetics.107.074278 17603105 PMC1950642

[B5] Nijman IJ Otsen M Verkaar ELC de Ruijter C Hanekamp E Ochieng JW Shamshad S Rege JEO Hanotte O Barwegen MW Sulawati T Lenstra JA Hybridization of banteng (Bos javanicus) and zebu (Bos indicus) revealed by mitochondrial DNA, satellite DNA, AFLP and microsatellites Hered 2003 90 10 16 10.1038/sj.hdy.6800174 12522420

[B6] Lecis R Pierpaoli M Biro ZS Szemethy L Ragni B Vercillo F Randi E Bayesian analyses of admixture in wild and domestic cats (Felis silvestris) using linked microsatellite loci Mol Ecol 2006 15 119 131 16367835 10.1111/j.1365-294X.2005.02812.x

[B7] Goodman SJ Barton NH Swanson G Abernethy K Pemberton JM Introgression through rare hybridization: a genetic study of a hybrid zone between red and sika deer (genus Cervus) in Argyll, Scotland Genetics 1999 152 355 371 10224266 10.1093/genetics/152.1.355 PMC1460577

[B8] Riley SPD Shaffer HB Voss SR Fitzpatrick BM Hybridization between a rare, native tiger salamander (Ambystoma californiense) and its introduced congener Ecol Applic 2003 13 1263 1275 10.1890/02-5023

[B9] Ropiquet A Hassanin A Hybrid origin of the Pliocene ancestor of wild goats Mol Phylogenet Evol 2006 41 395 404 10.1016/j.ympev.2006.05.033 16837213

[B10] Randi E Detecting hybridization between wild species and their domesticated relatives Mol Ecol 2008 17 285 293 10.1111/j.1365-294X.2007.03417.x 18173502

[B11] McGinnity P Prodoehl P Ferguson A Hynes R Maoiléidigh NO Baker N Cotter D O’Hea B Cooke D Rogan G Taggart T Cross T Fitness reduction and potential extinction of wild populations of Atlantic salmon, Salmo salar, as a result of interactions with escaped farm salmon Proc R Soc Lond B Biol Sci 2003 270 2443 2450 10.1098/rspb.2003.2520 PMC1691531 14667333

[B12] Hutchings JA Fraser DJ The nature of fisheries- and farming-induced evolution Mol Ecol 2008 17 294 313 10.1111/j.1365-294X.2007.03485.x 17784924

[B13] Wiley C Qvarnström A Gustafsson L Effects of hybridization on the immunity of collared Ficedula albicollis and pied flycatchers F. hypoleuca, and their infection by haemosporidians J Avian Biol 2009 40 352 357 10.1111/j.1600-048X.2009.04741.x

[B14] Simberloff D Hybridization between native and introduced wildlife species: importance for conservation Wildl Biol 1996 2 143 150

[B15] Hammer SE Schwammer HM Suchentrunk F Evidence for introgressive hybridization of captive markhor (Capra falconeri) with domestic goat: cautions for reintroduction Biochem Genet 2008 46 216 226 10.1007/s10528-008-9145-y 18228130

[B16] Pastorini J Zaramody A Curtis DJ Nievergelt CM Mundy NI Genetic analysis of hybridization and introgression between wild mongoose and brown lemurs BMC Evol Biol 2009 9 32 10.1186/1471-2148-9-32 19196458 PMC2657121

[B17] Cronin MA Mitochondrial DNA in wildlife taxonomy and conservation biology: cautionary notes Wildl Soc Bull 1993 21 339 348

[B18] Putze M Nürnberg S Fickel J Y-chromosomal markers for the European brown hare (Lepus europaeus, Pallas 1778) Eur J Wildl Res 2007 53 257 264 10.1007/s10344-007-0093-3

[B19] Fickel J Reinsch A Microsatellite markers for the European Roe deer (Capreolus capreolus) Mol Ecol 2000 9 994 995 10.1046/j.1365-294x.2000.00939-2.x 10886662

[B20] Ellegren H Microsatellites: simple sequences with complex evolution Nat Rev Genet 2004 5 435 445 15153996 10.1038/nrg1348

[B21] Barbara T Palma-Silva C Paggi GM Bered F Fay MF Lexer C Cross-species transfer of nuclear microsatellite markers: potential and limitations Mol Ecol 2007 16 3759 3767 10.1111/j.1365-294X.2007.03439.x 17850543

[B22] Pasqualotto AC Denning DW Anderson MJ A cautionary tale: lack of consistency in allele sizes between two laboratories for a published multilocus microsatellite typing system J Clin Microbiol 2007 45 522 528 10.1128/JCM.02136-06 17166958 PMC1829014

[B23] Klein J The Natural History of the Major Histocompatibility Complex 1986 Wiley, New York

[B24] Robinson J Waller MJ Parham P de Groot N Bontrop R Kennedy LJ Stoehr P Marsh SGE IMGT/HLA and IMGT/MHC: sequence databases for the study of the major histocompatibility complex Nucleic Acids Res 2003 31 311 314 10.1093/nar/gkg070 12520010 PMC165517

[B25] Hedrick PW Perspective: highly variable loci and their interpretation in evolution and conservation Evolution 1999 53 313 318 10.2307/2640768 28565409

[B26] Vilà C Seddon J Ellegren H Genes of domestic mammals augmented by backcrossing with wild ancestors Trends Genet 2005 21 214 218 10.1016/j.tig.2005.02.004 15797616

[B27] Mainguy J Worley K Côté SD Coltma DW Low MHC DRB class II diversity in the mountain goat: past bottlenecks and possible role of pathogens and parasites Conserv Genet 2007 8 885 891 10.1007/s10592-006-9243-5

[B28] Sommer S Schwab D Ganzhorn JU MHC diversity of endemic Malagasy rodents in relation to geographic range and social system Behav Ecol Sociobiol 2002 51 214 221 10.1007/s00265-001-0432-4

[B29] Murray BW Malik S White BN Sequence variation at the major histocompatibility complex locus DQϐ in Beluga whales (Delphinapterus leucas) Mol Biol Evol 1995 12 582 593 7659014 10.1093/oxfordjournals.molbev.a040238

[B30] Couturier MAJ The ibex in the Alpine Capra Aegagrus ibex ibex L. [in French] 1962 Imprimerie Allier, Grenoble 1564

[B31] Stüwe M Grodinsky C Reproductive biology of captive Alpine ibex (Capra ibex ibex L.) Zoo Biol 1987 6 331 339 10.1002/zoo.1430060407

[B32] Giacometti M Roganti R De Tann M Stahlberger-Saitbekova N Obexer-Ruff G Alpine ibex Capra ibex ibex x domestic goat C. aegagrus domestica hybrids in a restricted area of southern Switzerland Wildl Biol 2004 10 137 143

[B33] Moço G Guerreiro M Ferreira AF Rebelo A Loureiro A Petrucci-Fonseca F Pérez JM The ibex Capra pyrenaica returns to its former Portuguese range Oryx 2006 40 351 354 10.1017/S0030605306000718

[B34] Acevedo P Cassinello J Biology, ecology and status of Iberian ibex Capra pyrenaica: a critical review and research prospectus Mammal Rev 2009 39 17 32 10.1111/j.1365-2907.2008.00138.x

[B35] Alasaad S Soglia D Sarasa M Soriguer RC Pérez JM Granados JE Rasero R Zhu XQ Rossi L Skin-scale genetic structure of Sarcoptes scabiei populations from individual hosts: empirical evidence from Iberian ibex-derived mites Parasitol Res 2008 104 101 105 10.1007/s00436-008-1165-3 18758821

[B36] Sarasa M Serrano E Soriguer RC Grandos JE Fandos P Gonzalez G Joachim J Pérez JM Negative effect of the arthropod parasite, Sarcoptes scabiei, on testes mass in Iberian ibex, Capra pyrenaica Vet Parasitol 2011 175 306 312 10.1016/j.vetpar.2010.10.024 21074328

[B37] Amills M Jiménez N Jordana J Riccardi A Fernàndez-Arias A Guiral JL Bouzat J Folch J Sànchez A Low diversity in the major histocompatibility complex class II DRB1 gene of the Spanish ibex, Capra pyrenaica Heredity 2004 93 266 272 10.1038/sj.hdy.6800499 15241456

[B38] Luikart G Pilgrim K Visty J Ezenwa VO Schwartz MK Candidate gene microsatellite variation is associated with parasitism in wild bighorn sheep Biol Lett 2008 4 228 231 10.1098/rsbl.2007.0633 18270161 PMC2429941

[B39] Hall TA BioEdit: a user-friendly biological sequence alignment editor and analysis program for Windows 95/98/NT Nucleic Acids Symp Ser 1999 41 95 98

[B40] Schwaiger FW Buitkamp J Weyers E Epplen JT Typing of Artiodactyl MHC-DRB genes with the help of intronic simple repeated DRD-sequences Mol Ecol 1993 2 55 59 10.1111/j.1365-294X.1993.tb00099.x 8180732

[B41] Alasaad S Biebach I Grossen C Soriguer RC Pérez JM Keller LF DRB-STR matching method for Iberian and Alpine ibex MHC haplotyping Eur J Wildlife Res 2011 58 743 748

[B42] Takada T Kikkawa Y Yonekawa H Amano T Analysis of goat MHC class II DRA and DRB genes: identification of the expressed gene and new DRB alleles Immunogenetics 1998 48 408 412 10.1007/s002510050452 9799337

[B43] Richomme C Gauthier D Fromont E Contact rates and exposure to inter-species disease transmission in mountain ungulates Epidemiol Infect 2006 134 21 30 16409647 10.1017/S0950268805004693 PMC2870363

[B44] Gortázar C Acevedo P Ruiz-Fons F Vicente J Disease risks and overabundance of game species Eur J Wildl Res 2006 52 81 87 10.1007/s10344-005-0022-2

[B45] Acevedo P Cassinello J Gortázar C The Iberian ibex is under an expansion trend but displaced to suboptimal habitats by the presence of extensive goat livestock in central Spain Biodivers Conserv 2007 16 3361 3376 10.1007/s10531-006-9032-y

[B46] Rhymer JM Simberloff D Extinction by hybridization and introgression Annu Rev Ecol Syst 1996 27 83 109 10.1146/annurev.ecolsys.27.1.83

[B47] Sarasa M Pérez JM Alasaad S Serrano E Soriguer RC Granados JE Fandos P Joachim J Gonzalez G Neatness is a matter of season, age and sex in Iberian ibex Capra pyrenaica Behav Ecol 2011 22 1070 1078 10.1093/beheco/arr092

[B48] Schaschl H Suchentrunk F Hammer S Goodman SJ Recombination and the origin of sequence diversity in the DRB MHC class II locus in chamois (Rupicapra spp.) Immunogenet 2005 57 108 115 10.1007/s00251-005-0784-4 15756546

[B49] Schaschl H Wandeler P Suchentrunk F Obexer-Ruff G Goodman SJ Selection and recombination drive the evolution of MHC class II DRB diversity in ungulates Hered 2006 97 427 437 10.1038/sj.hdy.6800892 16941019

[B50] Bellemain E Taberlet P Improved noninvasive genotyping method: application to brown bear (Ursus arctos) faeces Mol Ecol Notes 2004 4 519 522 10.1111/j.1471-8286.2004.00711.x

