# Supplementary figures and images for: Applicability of major histocompatibility complex DRB1 alleles as markers to detect vertebrate hybridization: a case study from Iberian ibex × domestic goat in southern Spain
Source: Acta Vet Scand. 2012 Sep 24;54(1):56. doi: 10.1186/1751-0147-54-56 (PMC3511808; doi:10.1186/1751-0147-54-56)

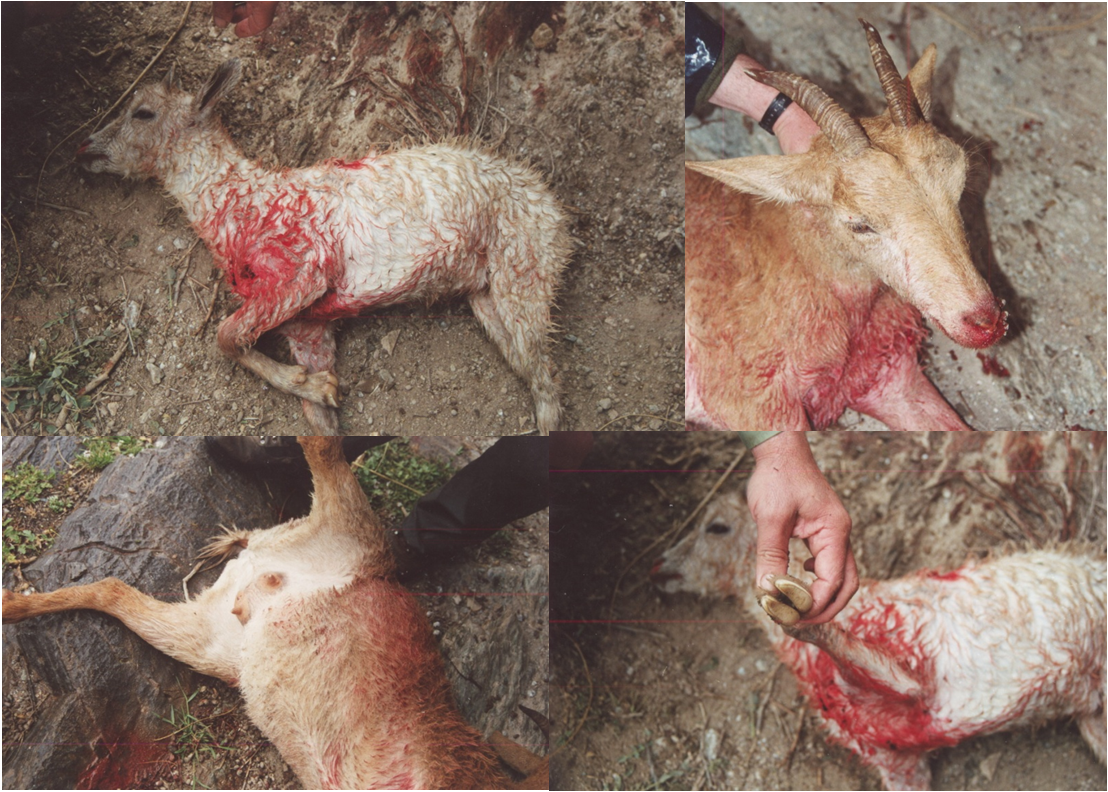

Supplement: Additional file 1 Figure S1. — Picture of Iberian ibex x domestic goat hybrid calf in the Mountain of Sierra Nevada in 1997. [file 1751-0147-54-56-S1.tiff]

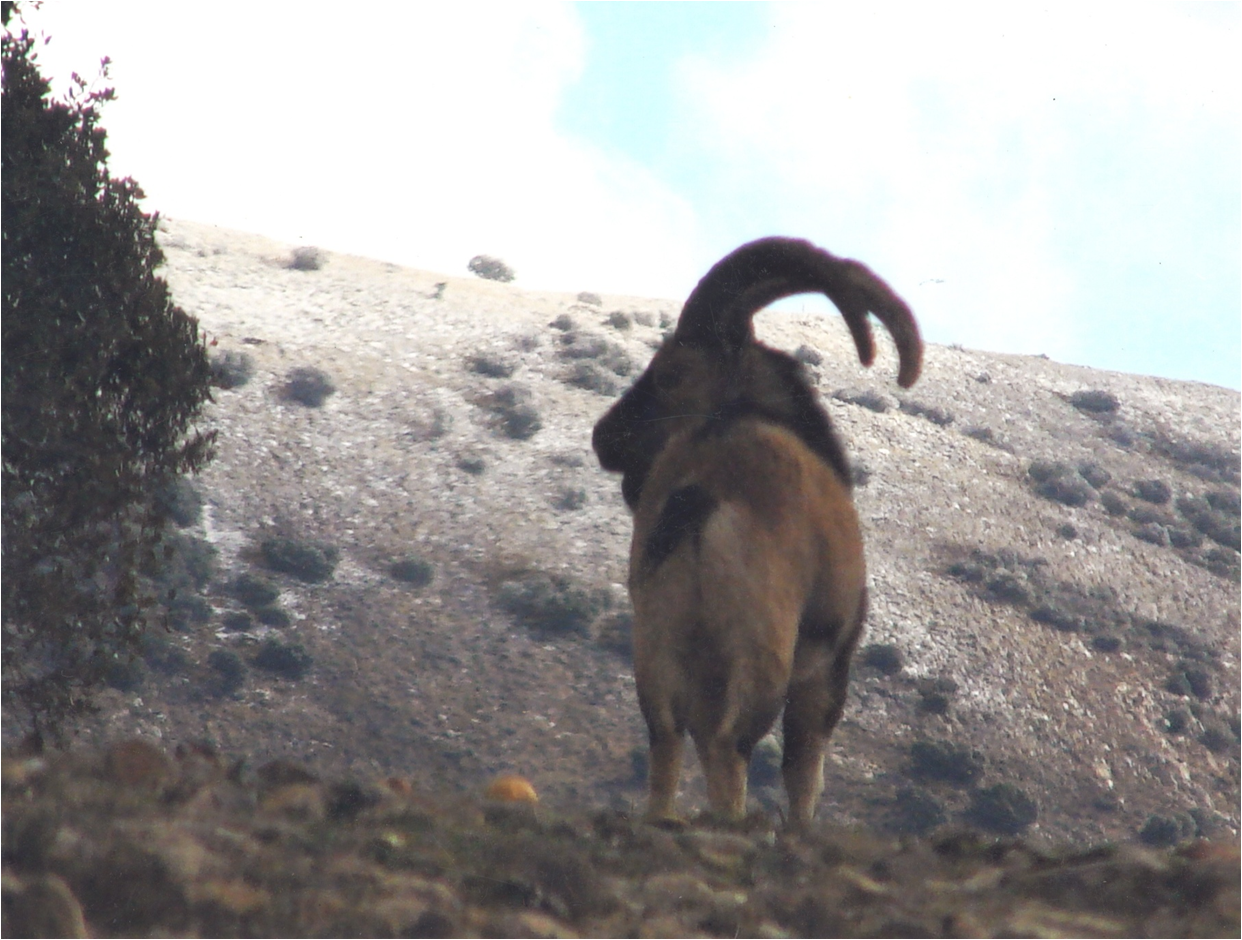

Supplement: Additional file 2 Figure S2. — Picture of Iberian ibex x domestic goat hybrid adult in the Mountain of Sierra Nevada in 1997. [file 1751-0147-54-56-S2.tiff]
